# Associations between hepatitis B virus exposure and the risk of extrahepatic digestive system cancers: A hospital‐based, case–control study (SIGES)

**DOI:** 10.1002/cam4.3901

**Published:** 2021-05-02

**Authors:** Hui Wang, Xin‐Zu Chen, Xiao‐Long Chen, Wei‐Han Zhang, Kai Liu, You‐Juan Wang, Huai‐Rong Tang, Jian‐Kun Hu, the SIGES research group

**Affiliations:** ^1^ Department of Gastrointestinal Surgery and Laboratory of Gastric Cancer State Key Laboratory of Biotherapy West China Hospital Sichuan University, and Collaborative Innovation Center for Biotherapy Chengdu China; ^2^ Department of Gastrointestinal Surgery The Central Hospital of Wuhan Tongji Medical College Huazhong University of Science and Technology Wuhan China; ^3^ Department of Gastrointestinal and Hernia Surgery Second People’s Hospital of Yibin City West China Yibin Hospital Sichuan University Yibin China; ^4^ Health Management Center West China Hospital Sichuan University Chengdu China

**Keywords:** case–control study, digestive system cancer, hepatitis B virus, infection, oncovirus

## Abstract

**Objectives:**

This case–control study was aimed to investigate associations between HBV infection and extrahepatic digestive system cancers.

**Methods:**

The patients of gastric, small intestinal, colonic, rectal, anal, biliary tract, and pancreatic cancers were retrospectively collected between 2016.5 and 2017.12. Simultaneously, the healthy controls were collected from the health check‐up registry, and cancer‐free status was confirmed based on medical records. Propensity score matching was performed to reduce bias. Multinomial logit model and conditional logistic regression model were used to assess the risk of individual cancer according to HBV serological markers and classifications.

**Results:**

Totally, 4748 patients involving seven cancers, and 57,499 controls were included. After matching, HBsAg was associated with increased risk of gastric cancer (aOR = 1.39, 95% CI: 1.05–1.85), and anti‐HBs served as a protective factor for gastric (aOR = 0.72, 95% CI: 0.61–0.85), colonic (aOR = 0.73, 95% CI: 0.60–0.89), rectal (aOR = 0.73, 95% CI: 0.63–0.85), and pancreatic (aOR = 0.58, 95% CI: 0.42–0.82) cancers. Compared to subgroups with non‐infection and vaccination status, inactive HBsAg carriers and active HBV infection subgroup were correlated with gastric carcinogenesis (aOR = 1.41, 95% CI: 1.03–1.93). However, no clear association was found between HBV infection and other cancers.

**Conclusions:**

HBV infection was potentially associated with an increased risk of gastric cancer. The development mechanism of HBV‐associated gastric cancer needs to investigate further.

## Graphical abstract

This case–control study was aimed to investigate associations between HBV infection and extrahepatic digestive system cancers. Our study revealed that HBV infection was potentially associated with an increased risk of gastric cancer. However, because of the limited ability to establish a causal relationship for case–control study, large‐scale prospective cohort studies are urgencies, and the latent mechanism about HBV extrahepatic carcinogenesis needs to be investigated as well.

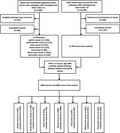

## 1 INTRODUCTION

It has been well known that several infectious oncoviruses are carcinogenic for specific human cancers.^1^, ^2^, ^3^ As one of eleven established carcinogenic agents (group 1) by the International Agency for Research on Cancer, hepatitis B virus (HBV) infection accounts for around 45% of cases of primary hepatocellular carcinoma (HCC).^4^, ^5^, ^6^ Its causal link to HCC has been investigated thoroughly and the natural history has been recognized. HBV carriers may progress to cirrhosis and HCC with chances.^7^ Since the existence of HBV was detected in some extrahepatic tissues (including pancreas, bile duct, stomach, etc.), several studies were conducted to illuminate a potential role in the development of extrahepatic cancers.^8^, ^9^, ^10^, ^11^, ^12^ Due to the blood vessels and bile ducts shared with the liver, the pancreas is vulnerable to viral hepatitis and serves as a potential reservoir of hepatitis viruses.^13^ Meanwhile, cholangiocarcinoma may share similar processes for HBV‐related carcinogenesis which originates from hepatic progenitor cells.^14^ Likewise, associations between HBV infection and various extrahepatic cancers were observed in several epidemiological studies which could be explained by the mobility of HBV through the bloodstream.^15^, ^16^, ^17^

As one of the HBV‐endemic areas, China is under a substantial disease burden resulted from HBV infection. Particularly, several studies implicated the HBV involvement in the onset of digestive system cancers including cholangiocarcinoma, pancreatic cancer, gastric cancer, small intestinal adenocarcinoma, etc.^18^, ^19^ However, the associations between HBV infection and the development of extrahepatic digestive system cancers were rarely described, and inconsistency was found because of the differences in study design, populations investigated, and the incidences of cancers or hepatitis.^20^, ^21^, ^22^ Compared to the well‐established association between HBV infection and HCC, the relationship between HBV infection and extrahepatic cancers in the abdominal digestive system was not systematically observed. Thus, we conducted this hospital‐based case–control study to investigate whether there were associations between HBV infection and extrahepatic digestive system cancers.

## 2 METHODS

### 2.1 Study population and eligibility

This hospital‐based case–control study was performed in the West China Hospital, Sichuan University, a joint with the **Si**chuan **G**astric Cancer **E**arly Detection and **S**creening (SIGES) research project.^23^ The patients with gastric, small intestinal, colonic, rectal, anal, biliary tract, and pancreatic cancers (*n* = 5105) were retrospectively searched according to the International Classification of Diseases‐10 (ICD‐10) codes from the electronic inpatient registry between 2016.5 and 2017.12. For patients with multiple cancers, only the first reported cancer type was included as the primary cancer. Included patient whose different cancers were recognized simultaneously was treated as different cases. For the same patients hospitalized repeatedly for the same cancer, only data of newly diagnosed were collected. The panel of HBV serology included hepatitis B surface antigen (HBsAg), antibody to HBsAg (anti‐HBs), hepatitis B e antigen (HBeAg), antibody to HBeAg (anti‐HBe), and antibody to hepatitis B core antigen (anti‐HBc). After excluding patients without pathological diagnosis and test results of the panel of HBV serology (*n* = 67), medical records were manually retrospective collected for consecutive hospitalized adult patients (≥18 years old) newly diagnosed with selected extrahepatic digestive system cancers (*n* = 5038). Anatomic locations of extrahepatic digestive system cancers were confirmed by ICD‐10 codes, imaging examination results, and/or surgical operation records. All patients of cancers were newly diagnosed with digestive system carcinoma according to pathologic examination, whereas patients with other histologic types, such as melanoma, sarcoma, lymphoma, gastrointestinal stromal tumor, and neuroendocrine neoplasm were excluded (*n* = 144).

During the same period, consecutive adult controls (≥18 years old, *n* = 59,496) who undergone HBV serological tests were collected from the health check‐up registry in the West China Hospital, Sichuan University. Their cancer‐free status was confirmed manually by the medical records including present and past medical history, and individuals with histories of cancer were excluded (*n* = 200).

**FIGURE 1:**
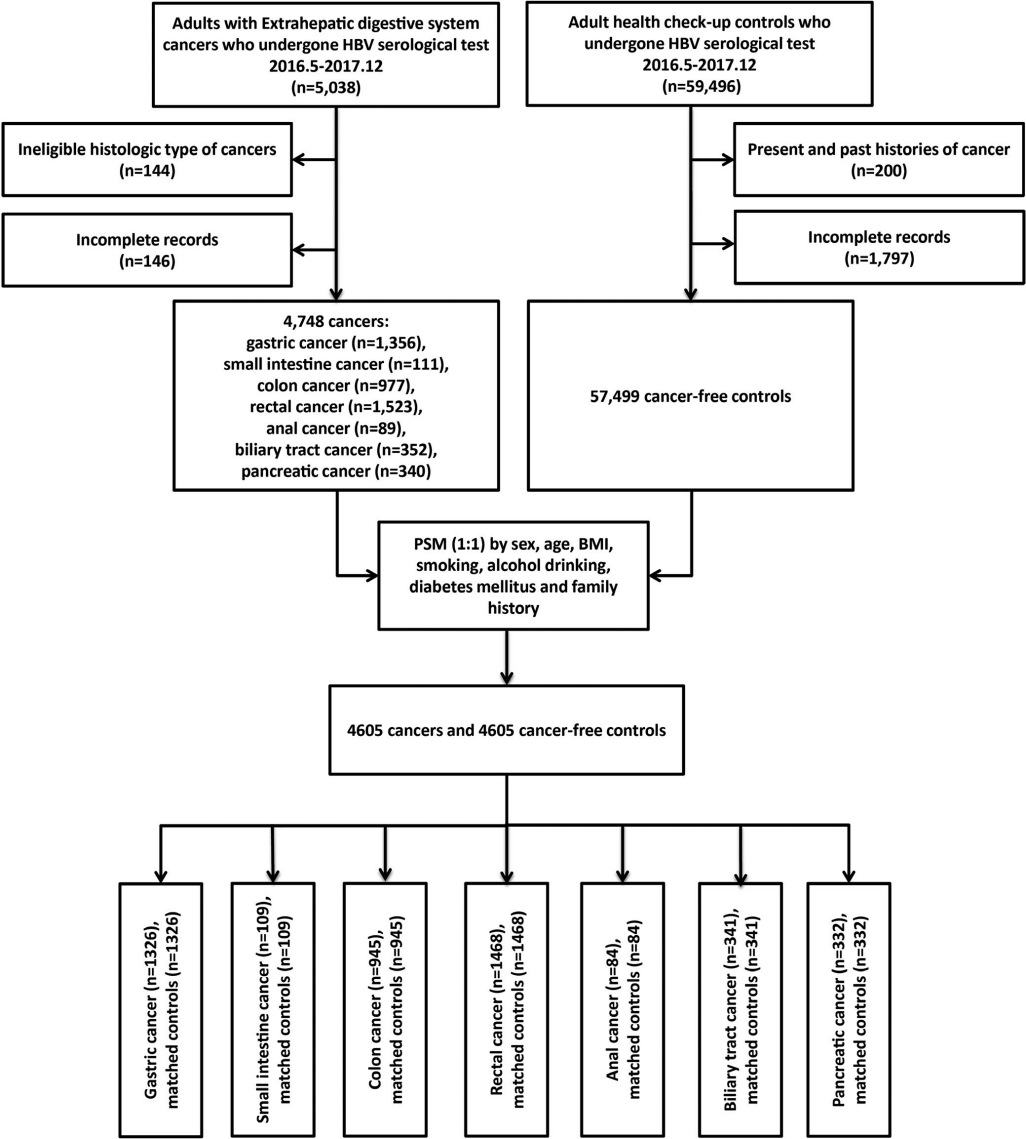
The flowchart of this hospital‐based case–control study

The information including sex, age, BMI, smoking status, alcohol drinking status, diabetes mellitus, family history of cancers, and results of HBV serology was collected in all observations. Any observation missing any medical record mentioned above was excluded in cancers (*n* = 146) and controls (*n* = 1797). Finally, 4748 inpatients of extrahepatic digestive system cancers including gastric (*n* = 1356), small intestinal (*n* = 111), colonic (*n* = 977), rectal (*n* = 1523), anal (*n* = 89), biliary tract (*n* = 352), and pancreatic cancer (*n* = 340), and 57,499 cancer‐free outpatients were included in this study (Figure 1).

### 2.2 Ethics

This hospital‐based case–control study retrospectively collected the information of baselines and serologic results of HBV markers. The SIGES study was approved by the Biomedical Ethical Committee of West China Hospital, Sichuan University (id: 2015–151‐V2, 2018–215‐V1). The informed consent was waived by the approval of the Biomedical Ethical Committee because of the retrospective nature. The private information was anonymized when analyzing and reporting data.

### 2.3 Laboratory tests

The presence of HBsAg in the blood indicates HBV infection. Following seroclearance of HBsAg, the appearance of anti‐HBs confers protection from HBV infection. Patients immunized after vaccination could be characterized by the presence of anti‐HBs as well. Seroprevalence of HBeAg reflects high levels of viral DNA replication and infectivity, and the presence of anti‐HBe represents host immune activation response to HBeAg and usually indicates decreasing HBV DNA and infectivity. Detection of anti‐HBc indicates previous HBV exposure.^24^, ^25^, ^26^ In our study, Electrochemiluminescence immunoassay (ECLIA) was used to test for the presence of five HBV‐related antibodies and antigens mentioned above. Meanwhile, tumor marker tests (including CA125, CA19‐9, CEA, and AFP) were performed for partial participants using ECLIA.

According to the WHO guideline and the clinical significance on the basis of HBV serology status, the observations were classified into three subgroups as follows: (a) Group A characterized by HBsAg–, HBeAg–, anti‐HBe– and anti‐HBc–, without or with anti‐HBs+ (non‐infection and vaccination); (b) Group B referred to HBsAg–/HBeAg–, and at least one of anti‐HBe+or anti‐HBc+, regardless of anti‐HBs+or not (resolved HBV infection); (c) Group C featured with HBsAg+/HBeAg±, regardless of the status of anti‐HBs, anti‐HBe, and anti‐HBc (inactive HBsAg carriers and active HBV infection).^24^, ^25^, ^26^

### 2.4 Statistical analysis

Three categories were classified on the basis of BMI: <23.0 kg/m^2^, 23.0–29.9 kg/m^2^, and ≥30.0 kg/m^2^.^27^ The Student's *t*‐test was used to compare continuous variables, and Fisher's exact test or Chi‐square test was used to assess categorical variables, respectively. 4748 patients in the case group were randomly matched with 57499 cancer‐free controls, and traditional propensity score matching was conducted to balance covariates (including sex, age, BMI, smoking status, alcohol drinking status, diabetes mellitus, and family history of cancers) and reduce bias, using a nearest‐neighbor algorithm by 1:1 matching with a caliper width of 0.25 (Figure 1).^28^ Standardized mean differences (SMDs) of covariates were estimated to evaluate the pre‐match imbalance and post‐match balance, and Love plots were presented and absolute SMDs<10% were considered inconsequential.^29^ Univariate or multivariate multinomial logit models were used to assess the risk associations between HBV serological markers or classifications and each cancer before matching. Re‐analyses based on matched datasets were conducted using a conditional logistic regression model for each cancer. Five HBV serological markers and three classifications were included for multivariate analyses adjusted by sex, age, BMI, smoking, alcohol drinking, diabetes mellitus, and family history of cancers. Adjusted odds ratios (aOR) and 95% confidence intervals (CIs) were estimated.

Data analyses were performed using the software R, version 3.6.0 (R Project for Statistical Computing), the multinomial logit model, propensity score matching, Love plots, conditional logistic model, and the forest plots were conducted with the packages of “mlogit,” “MatchIt,” “cobalt,” “survival,” and “forestplot.”^18^, ^30^, ^31^, ^32^, ^33^, ^34^ In all analyses, a two‐tailed *p* values <0.05 was considered as statistically significant.

## 3 RESULTS

### 3.1 Characteristics of study population

**FIGURE 2:**
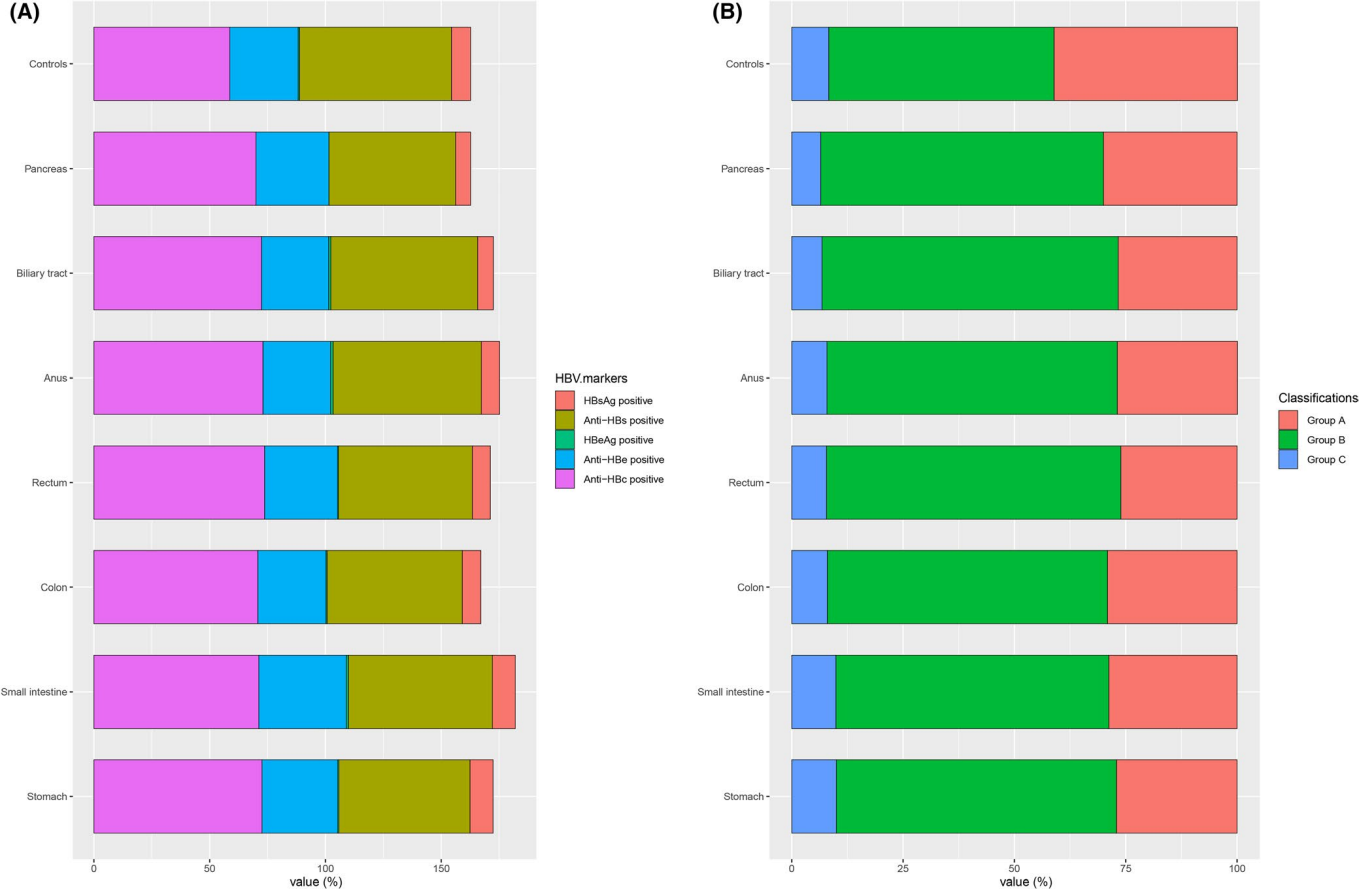
Positive rate of five HBV serological markers and three HBV serologic classifications by tumor site among cancer patients and non‐cancer subjects before matching. (A) Positive rate of five HBV serological markers; (B) Positive rate of three HBV serologic classifications

Basic characteristics of extrahepatic digestive system cancers and cancer‐free controls are shown in Table 1. Significant differences were observed between cancers and control populations in terms of sex, age, BMI, smoking status, alcohol drinking status, diabetes mellitus, and family history of cancers (*p *< 0.05). The seroprevalence of HBsAg was 8.4% in cancer cases and 8.3% in non‐cancer controls. As for individual cancer, serum positive rates of HBsAg in gastric, small intestinal, colonic, rectal, anal, biliary tract, and pancreatic cancers were 10.0%, 9.9%, 8.0%, 7.8%, 7.9%, 6.8%, and 6.5%, respectively, and the seroprevalence of anti‐HBc hold the largest proportion in all seven cancers. Similarly, most subjects both in each cancer case and controls were Group B (resolved HBV infection) (Table S1 and Figure 2).

**TABLE 1:** Basic characteristics of extrahepatic digestive system cancers and cancer‐free controls before and after matching

Variables	Unmatched dataset			Matched dataset		
	Cancers (n = 4748) No. (%)	Controls (n = 57499) No. (%)	*p*	Cancers (n = 4605) No. (%)	Controls (n = 4605) No. (%)	*p*
Sex			<0.001			0.949
Female	1824 (38.4)	27141 (47.2)		1805 (39.2)	1801 (39.1)	
Male	2924 (61.6)	30358 (52.8)		2800 (60.8)	2804 (60.9)	
Age (mean ± SD), years	59.99±12.32	45.01±12.16	<0.001	59.40±12.00	58.83±12.96	0.026
BMI (kg/m^2^)			<0.001			0.772
<23.0	2689 (56.6)	25408 (44.2)		2548 (55.3)	2516 (54.6)	
23.0–29.9	1972 (41.5)	30232 (52.6)		1971 (42.8)	2005 (43.5)	
≥30.0	87 (1.8)	1859 (3.2)		86 (1.9)	84 (1.8)	
Smoker			<0.001			0.135
Never	2916 (61.4)	43951 (76.4)		2888 (62.7)	2958 (64.2)	
Previous/Current	1832 (38.6)	13548 (23.6)		1717 (37.3)	1647 (35.8)	
Alcohol drinker			<0.001			0.535
Never	3969 (83.6)	51028 (88.7)		3842 (83.4)	3865 (83.9)	
Previous/Current	779 (16.4)	6471 (11.3)		763 (16.6)	740 (16.1)	
Diabetes mellitus (yes)	167 (3.5)	1490 (2.6)	<0.001	166 (3.6)	158 (3.4)	0.692
Family history of cancers (yes)	667 (14.0)	7419 (12.9)	0.026	651 (14.1)	615 (13.4)	0.290
HBV markers						
HBsAg positive	397 (8.4)	4752 (8.3)	0.837	389 (8.4)	336 (7.3)	0.044
Anti‐HBs positive	2754 (58.0)	37727 (65.6)	<0.001	2667 (57.9)	3022 (65.6)	<0.001
HBeAg positive	23 (0.5)	333 (0.6)	0.464	23 (0.5)	11 (0.2)	0.059
Anti‐HBe positive	1488 (31.3)	16968 (29.5)	0.008	1439 (31.2)	1461 (31.7)	0.638
Anti‐HBc positive	3437 (72.4)	33763 (58.7)	<0.001	3318 (72.1)	3255 (70.7)	0.153
Classifications^a^			<0.001			0.057
Group A	1300 (27.4)	23669 (41.2)		1276 (27.7)	1345 (29.2)	
Group B	3051 (64.3)	29078 (50.6)		2940 (63.8)	2924 (63.5)	
Group C	397 (8.4)	4752 (8.3)		389 (8.4)	336 (7.3)	

^a^ (1) Group A characterized by HBsAg–, HBeAg–, anti‐HBe– and anti‐HBc–, without or with anti‐HBs+ (non‐infection and vaccination); (2) Group B referred to HBsAg–/HBeAg–, and at least one of anti‐HBe+or anti‐HBc+, regardless of anti‐HBs+or not (resolved HBV infection); (3) Group C featured with HBsAg+/HBeAg±, regardless of the status of anti‐HBs, anti‐HBe and anti‐HBc (inactive HBsAg carriers and active HBV infection).

**FIGURE 3:**
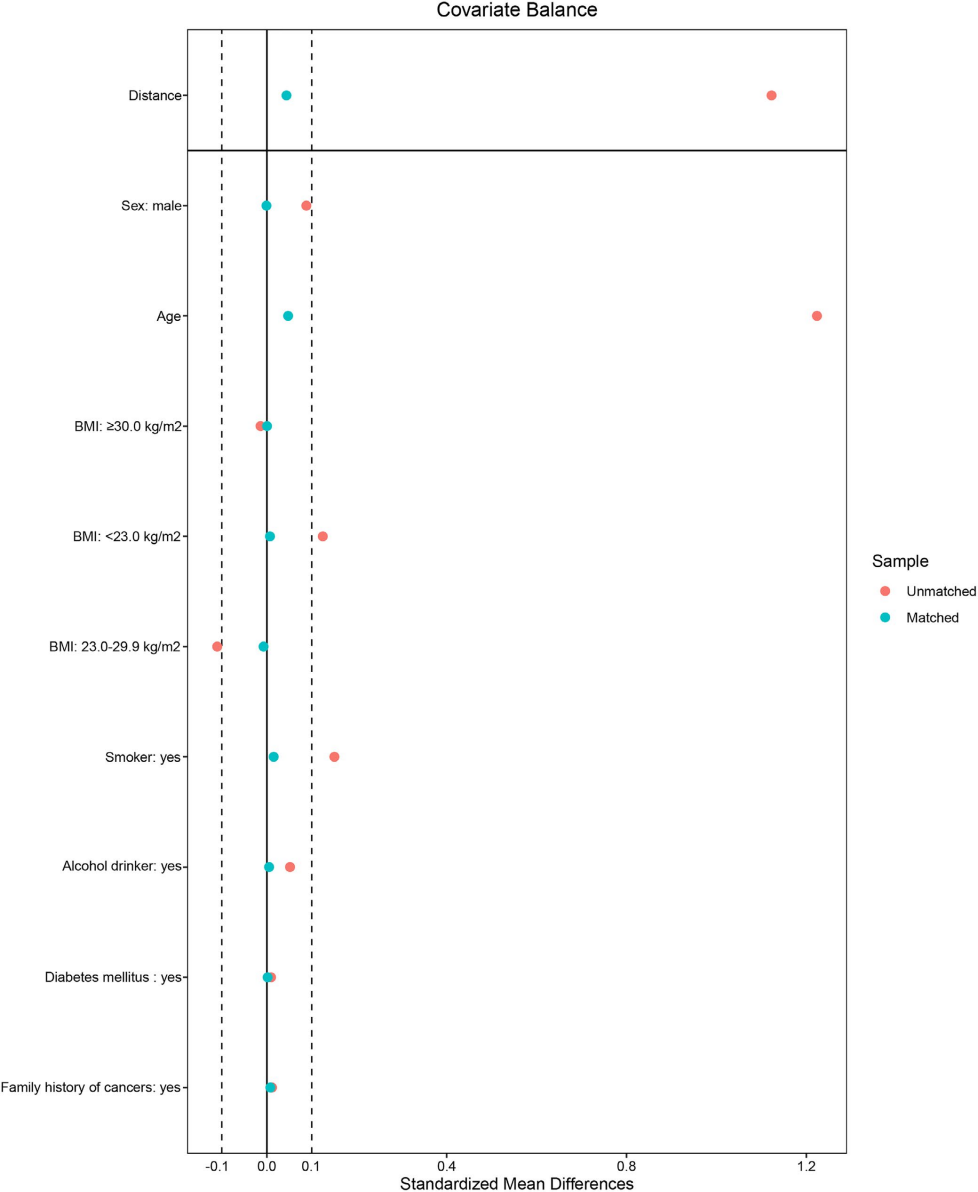
Standardized mean differences of covariates before and after matching

After matching, there was no substantial difference between cancer cases and controls in terms of sex, BMI, smoking status, alcohol drinking status, diabetes mellitus, and family history of cancers (*p *> 0.05) (Table 1 and Figure 3). As for individual cancer, the seroprevalence of HBsAg was higher only in gastric cancer compared with matched controls (*p* = 0.017). A significant difference between gastric cancer and controls was also observed in terms of HBV serologic classifications (*p* = 0.036). In addition, the serum positive rates of anti‐HBs were lower in gastric, colonic, rectal, and pancreatic cancers than those in matched controls (*p *< 0.05) (Table S2).

### 3.2 Associations between HBV serology status and each extrahepatic digestive system cancer before matching

In univariate analysis, HBsAg (OR = 1.24, 95% CI: 1.03–1.48) and anti‐HBe (OR = 1.16, 95% CI: 1.04–1.30) were associated with increased risk of gastric cancer only. Anti‐HBc was the risk factor for all seven cancers. On the contrary, anti‐HBs was statistically associated with decreased risk of gastric (OR = 0.68, 95% CI: 0.61–0.76), colonic (OR = 0.74, 95% CI: 0.65–0.84), rectal (OR = 0.72, 95% CI: 0.65–0.80), and pancreatic (OR = 0.63, 95% CI: 0.51–0.78) cancers. In terms of HBV serologic classifications, the prevalence of group B in all seven cancers and group C in gastric, colonic, rectal cancers were significantly higher compared with group A (Table S3).

**FIGURE 4:**
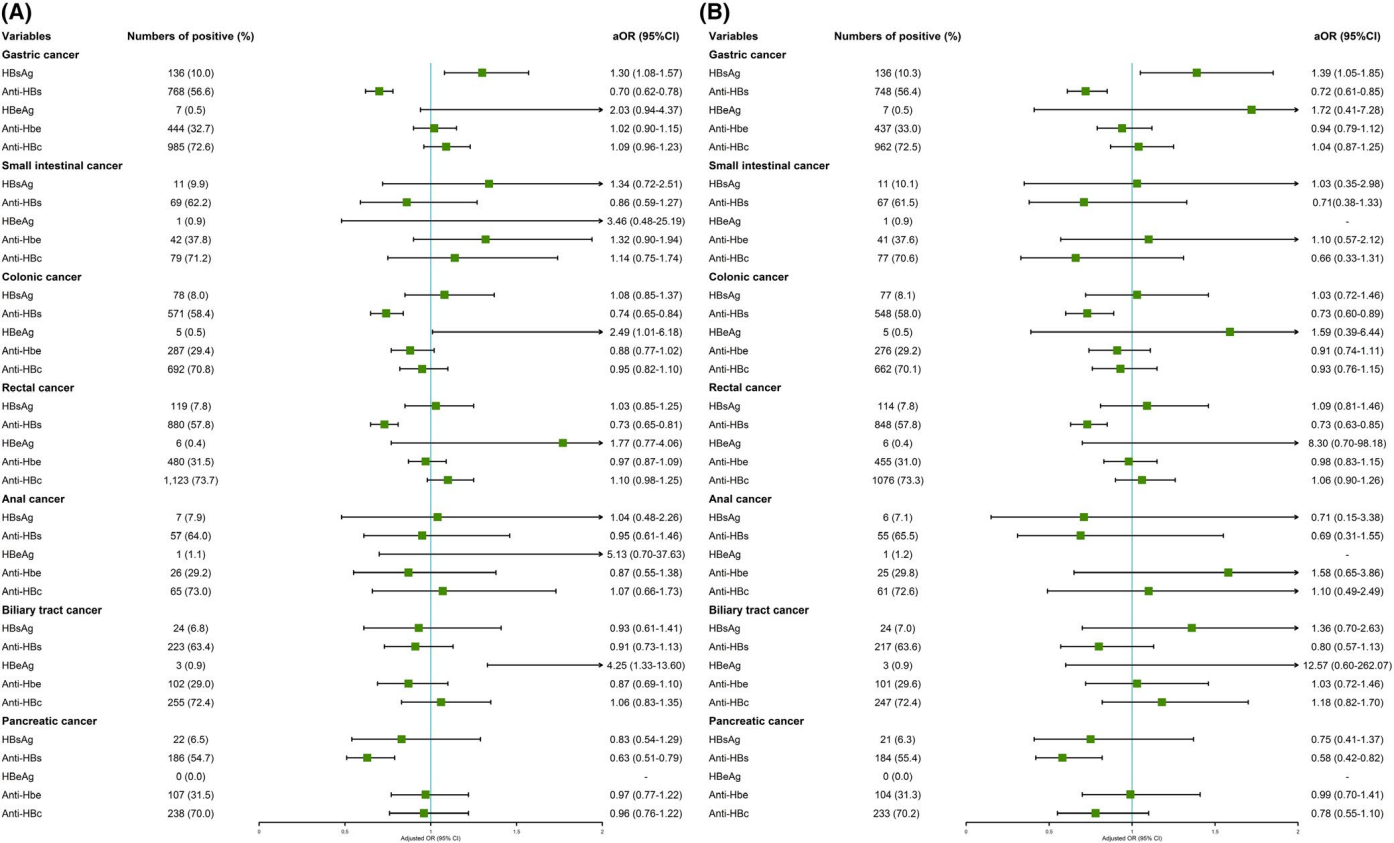
The subgroup analyses on cancer risk by five HBV serological markers (multivariate analyses adjusted sex, age, BMI, smoking status, alcohol drinking status, diabetes mellitus, and family history of cancers) (A) before matching; (B) after matching

**FIGURE 5:**
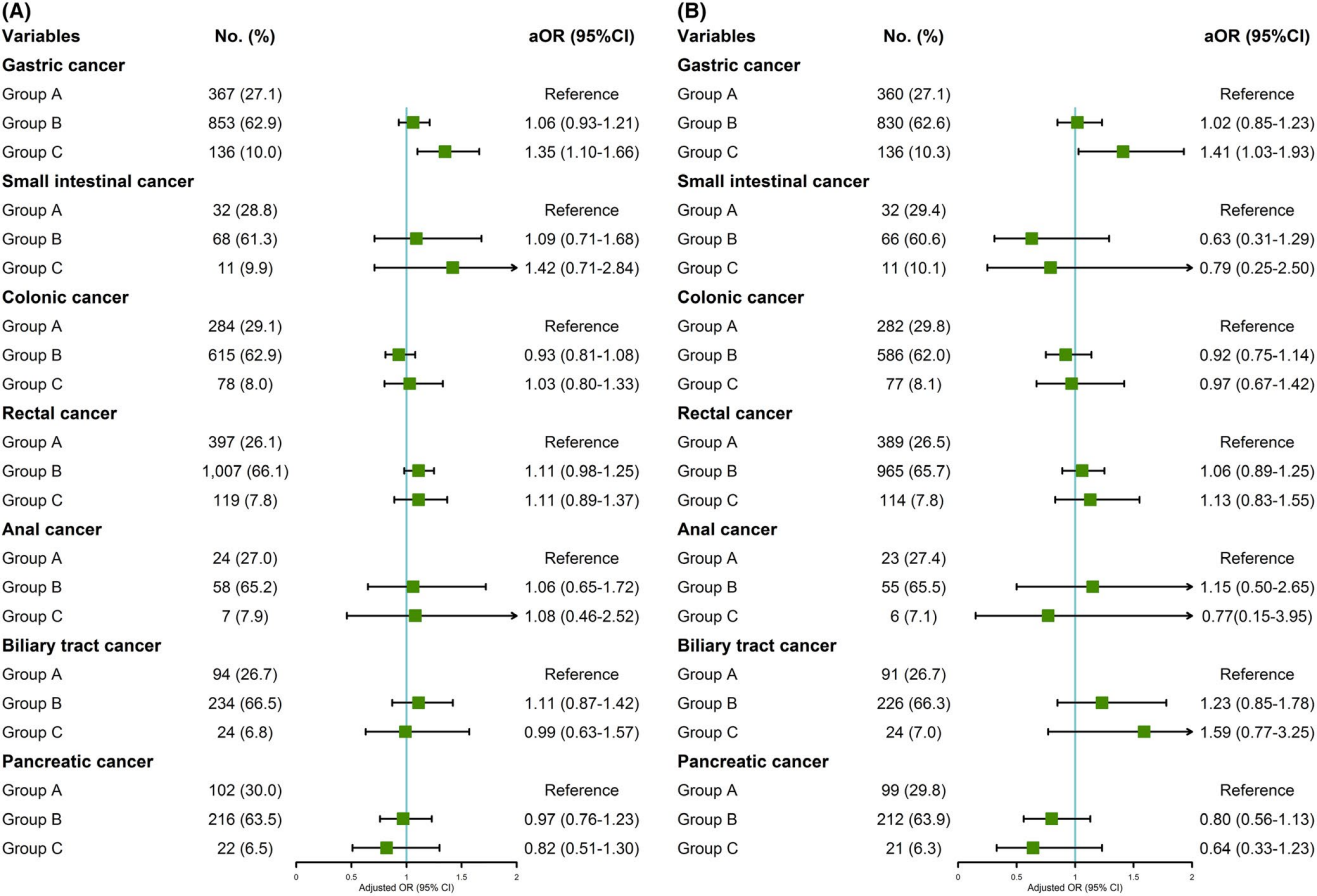
The subgroup analyses on cancer risk by three HBV serologic classifications (multivariate analyses adjusted sex, age, BMI, smoking status, alcohol drinking status, diabetes mellitus, and family history of cancers) (A) before matching; (B) after matching

In multivariable analysis, there was a significant association between HBsAg positivity and gastric cancer compared with cancer‐free controls (aOR = 1.30, 95% CI: 1.08–1.57), and HBeAg was the risk factor for colonic (aOR = 2.49, 95% CI: 1.01–6.18) and biliary tract (aOR = 4.25, 95% CI: 1.33–13.60) cancers. Similarly, anti‐HBs was statistically associated with decreased risk of gastric (aOR = 0.70, 95% CI: 0.62–0.78), colonic (aOR = 0.74, 95% CI: 0.65–0.84), rectal (aOR = 0.73, 95% CI: 0.65–0.81), and pancreatic (aOR = 0.63, 95% CI: 0.51–0.79) cancers. In terms of HBV serologic classifications, the prevalence of group C in gastric cancer was significantly higher compared with group A (aOR = 1.35, 95% CI: 1.10–1.66) (Table 2, Figure 4A and Figure 5A).

**TABLE 2:** Associations between HBV serology status and extrahepatic digestive system cancers: multivariate analyses adjusted by age, sex, BMI, smoking, alcohol drinking, diabetes mellitus, and family history of cancers according to tumor location before and after matching

Variables	Unmatched datasets			Matched datasets		
	aOR	95% CI	*p*	aOR	95% CI	*p*
Gastric cancer						
HBsAg (+/−)	1.30	1.08–1.57	0.005	1.39	1.05–1.85	0.024
Anti‐HBs (+/−)	0.70	0.62–0.78	<0.001	0.72	0.61–0.85	<0.001
HBeAg (+/−)	2.03	0.94–4.37	0.072	1.72	0.41–7.28	0.461
Anti‐HBe (+/−)	1.02	0.90–1.15	0.770	0.94	0.79–1.12	0.496
Anti‐HBc (+/−)	1.09	0.96–1.23	0.190	1.04	0.87–1.25	0.660
Classifications^a^						
Group A	1.00	Reference		1.00	Reference	
Group B	1.06	0.93–1.21	0.376	1.02	0.85–1.23	0.846
Group C	1.35	1.10–1.66	0.004	1.41	1.03–1.93	0.034
Small intestinal cancer						
HBsAg (+/−)	1.34	0.72–2.51	0.355	1.03	0.35–2.98	0.960
Anti‐HBs (+/−)	0.86	0.59–1.27	0.458	0.71	0.38–1.33	0.284
HBeAg (+/−)	3.46	0.48–25.19	0.220	—	—	—
Anti‐HBe (+/−)	1.32	0.90–1.94	0.156	1.10	0.57–2.12	0.786
Anti‐HBc (+/−)	1.14	0.75–1.74	0.543	0.66	0.33–1.31	0.230
Classifications^a^						
Group A	1.00	Reference		1.00	Reference	
Group B	1.09	0.71–1.68	0.692	0.63	0.31–1.29	0.207
Group C	1.42	0.71–2.84	0.316	0.79	0.25–2.50	0.690
Colonic cancer						
HBsAg (+/−)	1.08	0.85–1.37	0.524	1.03	0.72–1.46	0.878
Anti‐HBs (+/−)	0.74	0.65–0.84	<0.001	0.73	0.60–0.89	0.002
HBeAg (+/−)	2.49	1.01–6.18	0.048	1.59	0.39–6.44	0.518
Anti‐HBe (+/−)	0.88	0.77–1.02	0.088	0.91	0.74–1.11	0.352
Anti‐HBc (+/−)	0.95	0.82–1.10	0.471	0.93	0.76–1.15	0.520
Classifications^a^						
Group A	1.00	Reference		1.00	Reference	
Group B	0.93	0.81–1.08	0.360	0.92	0.75–1.14	0.457
Group C	1.03	0.80–1.33	0.820	0.97	0.67–1.42	0.892
Rectal cancer						
HBsAg (+/−)	1.03	0.85–1.25	0.764	1.09	0.81–1.46	0.563
Anti‐HBs (+/−)	0.73	0.65–0.81	<0.001	0.73	0.63–0.85	<0.001
HBeAg (+/−)	1.77	0.77–4.06	0.176	8.30	0.70–98.18	0.093
Anti‐HBe (+/−)	0.97	0.87–1.09	0.600	0.98	0.83–1.15	0.801
Anti‐HBc (+/−)	1.10	0.98–1.25	0.105	1.06	0.90–1.26	0.487
Classifications^a^						
Group A	1.00	Reference		1.00	Reference	
Group B	1.11	0.98–1.25	0.106	1.06	0.89–1.25	0.541
Group C	1.11	0.89–1.37	0.358	1.13	0.83–1.55	0.444
Anal cancer						
HBsAg (+/−)	1.04	0.48–2.26	0.918	0.71	0.15–3.38	0.662
Anti‐HBs (+/−)	0.95	0.61–1.46	0.807	0.69	0.31–1.55	0.371
HBeAg (+/−)	5.13	0.70–37.63	0.108	—	—	—
Anti‐HBe (+/−)	0.87	0.55–1.38	0.558	1.58	0.65–3.86	0.318
Anti‐HBc (+/−)	1.07	0.66–1.73	0.780	1.10	0.49–2.49	0.816
Classifications^a^						
Group A	1.00	Reference		1.00	Reference	
Group B	1.06	0.65–1.72	0.821	1.15	0.50–2.65	0.738
Group C	1.08	0.46–2.52	0.855	0.77	0.15–3.95	0.750
Biliary tract cancer						
HBsAg (+/−)	0.93	0.61–1.41	0.721	1.36	0.70–2.63	0.362
Anti‐HBs (+/−)	0.91	0.73–1.13	0.379	0.80	0.57–1.13	0.204
HBeAg (+/−)	4.25	1.33–13.60	0.015	12.57	0.60–262.07	0.102
Anti‐HBe (+/−)	0.87	0.69–1.10	0.247	1.03	0.72–1.46	0.885
Anti‐HBc (+/−)	1.06	0.83–1.35	0.644	1.18	0.82–1.70	0.360
Classifications^a^						
Group A	1.00	Reference		1.00	Reference	
Group B	1.11	0.87–1.42	0.414	1.23	0.85–1.78	0.278
Group C	0.99	0.63–1.57	0.980	1.59	0.77–3.25	0.208
Pancreatic cancer						
HBsAg (+/−)	0.83	0.54–1.29	0.418	0.75	0.41–1.37	0.344
Anti‐HBs (+/−)	0.63	0.51–0.79	<0.001	0.58	0.42–0.82	0.002
HBeAg (+/−)	—	—	—	—	—	—
Anti‐HBe (+/−)	0.97	0.77–1.22	0.781	0.99	0.70–1.41	0.964
Anti‐HBc	0.96	0.76–1.22	0.739	0.78	0.55–1.10	0.155
Classifications^a^						
Group A	1.00	Reference		1.00	Reference	
Group B	0.97	0.76–1.23	0.795	0.80	0.56–1.13	0.202
Group C	0.82	0.51–1.30	0.394	0.64	0.33–1.23	0.176

^a^ (1) Group A characterized by HBsAg–, HBeAg–, anti‐HBe– and anti‐HBc–, without or with anti‐HBs+ (non‐infection and vaccination); (2) Group B referred to HBsAg–/HBeAg–, and at least one of anti‐HBe+or anti‐HBc+, regardless of anti‐HBs+or not (resolved HBV infection); (3) Group C featured with HBsAg+/HBeAg±, regardless of the status of anti‐HBs, anti‐HBe and anti‐HBc (inactive HBsAg carriers and active HBV infection).

### 3.3 Re‐analyses after matching

Re‐analyses based on matched datasets were conducted. In univariate analysis, HBsAg remained the positive association with gastric cancer (OR = 1.41, 95% CI: 1.07–1.85). Similarly, anti‐HBs still was a protective factor for gastric (OR = 0.73, 95% CI: 0.62–0.85), colonic (OR = 0.72, 95% CI: 0.60–0.88), rectal (OR = 0.71, 95% CI: 0.62–0.83), and pancreatic (OR = 0.58, 95% CI: 0.42–0.80) cancers. In terms of HBV serologic classifications, compared with group A, group C was associated with an increased risk of gastric cancer (OR = 1.49, 95% CI: 1.10–2.02) (Table S3).

In multivariable analysis, HBsAg was still associated with increased risk of gastric cancer (aOR = 1.39, 95% CI: 1.05–1.85), anti‐HBs was also associated with decreased risk of gastric (aOR = 0.72, 95% CI: 0.61–0.85), colonic (aOR = 0.73, 95% CI: 0.60–0.89), rectal (aOR = 0.73, 95% CI: 0.63–0.85), and pancreatic (aOR = 0.58, 95% CI: 0.42–0.82) cancers, group C (aOR = 1.41, 95% CI: 1.03–1.93) was associated with an increased risk of gastric cancer compared with group A in terms of HBV serologic classifications. Results mentioned above were consistent with results before matching (Table 2, Figure 4B and Figure 5B).

### 3.4 Associations between risk factors and HBsAg positive in gastric cancer

We have observed a positive correlation trend between HBsAg and gastric cancer before and after matching, associations between risk factors and HBsAg positive in gastric cancer were analyzed based on the above conclusions. Four serum tumor markers including CA125, CA19‐9, CEA, and AFP were tested in 1161 of 1356 gastric cancer patients. Significant differences were observed between gastric cancer patients with and without HBsAg positive in terms of sex, age, family history of cancers, and AFP level (*p *< 0.05). Both in univariate and multivariable unconditional logistic regression model, sex, age, and AFP level were correlated with HBsAg in gastric cancer. Compared to HBsAg– patients, HBsAg+patients were younger which had a higher prevalence of abnormal AFP level (≥8 ng/ml) and a higher proportion of males (Table 3).

**TABLE 3:** Basic characteristics of patients with and without HBsAg positive, associations between risk factors and HBsAg positive in gastric cancer

Variables	HBsAg positive (n = 115) No. (%)	HBsAg negative (*n* = 1046) No. (%)	*p*	Univariate analysis			Multivariate analysis		
				OR	95% CI	*p*	aOR	95% CI	*p*
Sex			0.021						
Female	28 (24.3)	372 (35.6)		1.00	Reference		1.00	Reference	
Male	87 (75.7)	674 (64.4)		1.71	1.10–2.67	0.017	2.00	1.14–3.52	0.016
Age (mean ±SD), years	55.82±10.63	58.60±11.61	0.014	0.98	0.96–1.00	0.014	0.97	0.95–0.99	0.001
BMI (kg/m^2^)			0.190						
<23.0	59 (51.3)	627 (59.9)		1.00	Reference		1.00	Reference	
23.0–29.9	54 (47.0)	400 (38.2)		1.43	0.97–2.12	0.070	1.43	0.95–2.14	0.083
≥30.0	2 (1.7)	19 (1.8)		1.12	0.25–4.92	0.882	1.07	0.24–4.79	0.933
Smoker			0.229						
Never	57 (49.6)	585 (55.9)		1.00	Reference		1.00	Reference	
Previous/Current	58 (50.4)	461 (44.1)		1.29	0.88–1.90	0.194	0.87	0.53–1.42	0.579
Alcohol drinker			0.217						
Never	88 (76.5)	855 (81.7)		1.00	Reference		1.00	Reference	
Previous/Current	27 (23.5)	191 (18.3)		1.37	0.87–2.17	0.175	1.32	0.79–2.20	0.296
Diabetes mellitus (yes)	4 (3.5)	30 (2.9)	0.939	1.22	0.42–3.53	0.713	1.10	0.37–3.29	0.859
Family history of cancers (yes)	25 (21.7)	150 (14.3)	0.049	1.66	1.03–2.67	0.037	1.63	0.99–2.67	0.053
CA125 (≥35 U/ml)	8 (7.0)	69 (6.6)	1.000	1.06	0.50–2.26	0.883	0.99	0.45–2.19	0.981
CA19‐9 (≥22 U/ml)	32 (27.8)	231 (22.1)	0.201	1.36	0.88–2.10	0.164	1.52	0.96–2.41	0.076
CEA (≥3.4 ng/ml)	33 (28.7)	288 (27.5)	0.877	1.06	0.69–1.62	0.791	0.96	0.61–1.51	0.854
AFP (≥8 ng/ml)	14 (12.2)	54 (5.2)	0.005	2.55	1.37–4.75	0.003	2.28	1.20–4.34	0.012

## 4 DISCUSSION

As one of HBV serological markers, the presence of detectable HBsAg indicated HBV infection. Following seroclearance of HBsAg, the appearance of anti‐HBs confers protection from HBV infection.^24^ Our study revealed that HBsAg was associated with an increased risk of gastric cancer and anti‐HBs served as a protective factor for gastric, colonic, rectal, and pancreatic cancers. Compared to subgroups with non‐infection and vaccination status, inactive HBsAg carriers and active HBV infection subgroup were correlated with gastric carcinogenesis.

China is one of the HBV‐endemic areas and the prevalence of HBsAg carriers experienced a decline from 9.8% to 7.2% during 1992–2006 due to national immunization.^35^, ^36^, ^37^ However, the majority of HBV infection develops into persistent and chronic viral infection more easily by reason of early onset age and low spontaneous clearance rate of HBsAg.^36^, ^37^ In our study, the serum positive rates of HBsAg was 8.4% (6.5%‐10.0%) in cancer cases and 8.3% in non‐cancer controls, respectively, slightly above national average rate (around 8%).^38^, ^39^

Since overexpression of HBsAg and HBcAg was detected in gastric epithelial cells, several previous studies sought to investigate the association between HBV infection and gastric cancer with conflicting results.^11^, ^15^, ^16^, ^18^, ^22^, ^40^, ^41^, ^42^ As precancerosis to gastric cancer, gastric premalignant changes was found to be correlated with HBV infection.^22^ Meta‐analyses also found the association between HBV infection and gastric cancer.^43^, ^44^ In our study, the seroprevalence of HBsAg was much higher in gastric cancer compared with matched controls (*p* = 0.017). We verified that HBsAg was significantly associated with increased risk of gastric cancer independently.^18^, ^41^, ^42^ Meanwhile, for patients with gastric cancer, HBsAg+patients were younger and had a higher proportion of male compared with HBsAg– patients.^45^

In addition to gastric cancer, a higher incidence of colorectal adenoma was also associated with HBV infection.^46^ As precancerosis to colorectal cancer, colorectal adenoma can progress to colorectal carcinoma with chances. For pancreatic cancer, its association with HBV infection was controversial.^13^, ^20^, ^21^, ^47^, ^48^, ^49^ Statistically significant association between patients with past exposure to HBV and the risk of pancreatic cancer was observed in Hassan's study not in Tang's.^13^, ^50^ In our study, 340 patients with pancreatic cancer were included and the serum‐positive rates of HBsAg were 6.5% (22/340) which was lower than Tian's (7.2%, 146/2039).^49^ Non‐significant association between HBsAg positivity and pancreatic cancer was observed that may be due to the small sample size in our study. Furthermore, the different prevalence of HBV and pancreatic cancer by different geographic regions investigated, year of pancreatic cancer diagnosis, and treatment of confounding factors might explain this discrepancy in results.^49^ It was also found that HBV was an independent factor in the risk for cholangiocarcinoma.^51^, ^52^, ^53^, ^54^, ^55^, ^56^ However, no clear association was found between HBV infection and other cancers except gastric cancer in our study. Interestingly, our study revealed that anti‐HBs were statistically associated with decreased risk of gastric, colonic, rectal, and pancreatic cancers. As a protecting antibody arising after HBsAg, anti‐HBs was associated with decreased risk of cancers mentioned above perhaps signified the positive associations between HBsAg and cancers mentioned above.^42^

HBV has been recognized as a causative pathogen for HCC which had high incidence and mortality in China. It has been suggested that HBV trigger the host's immune responses to create a hypoxic environment for supporting virus persistent replication and prolonging chronic inflammation without virus clearance, which causes the integration of viral encoded proteins into human chromosomes and the mutation of host gene expression and cellular phenotypes that confer the pathogenesis of HCC.^57^

As a transactivating protein encoded by HBV, hepatitis B X (HBX) protein is associated with initiating the development of HCC. The expression of HBX and anti‐HBc was detected in gastric cancer which indicated persistent and chronic viral infection.^11^, ^18^, ^41^, ^42^ Chronic inflammation triggered by HBV caused immunosuppression which might play a role in gastric carcinogenesis. Cui et al found cellular atypia and lymphocytes’ infiltration induced by HBX in gastric epithelial cells.^44^ As the oncogenic protein, HBX might also involve in the development of gastric cancer. For patients with gastric cancer, the proportion of AFP abnormal elevation was much higher in HBsAg+patients compared with HBsAg– patients which indicated a similar mechanism of HBV‐related HCC exists in gastric carcinogenesis.

It has been reported that HBX competitively binds adenomatous polyposis coli (APC) to activate Wnt/b‐catenin signaling, and then induces hallmark changes of cancer further.^58^ Wnt/b‐catenin signaling was also identified as a related signaling pathway to colorectal carcinogenesis. For this reason, it was speculated that a similar mechanism may exist in colorectal cancer.^46^ Iloeje et al considered that HBV persistent replication might exist in the pancreas which is vulnerable to viral hepatitis and serves as a potential reservoir of hepatitis viruses. Cell injury, immunoreaction, and inflammation caused by chronic HBV infection might play an important role in pancreatic carcinogenesis.^47^, ^59^ Like the pancreas adjacent to the liver, cholangiocarcinoma may share similar processes for HBV‐related carcinogenesis which originate from hepatic progenitor cells, and chronic inflammatory process might involve in the development of cholangiocarcinoma as well.^14^, ^60^, ^61^

Compared with previous studies, the relationship between HBV infection and the risk of extrahepatic digestive system cancers was systematically examined in our study. Unlike several previous studies which only defined HBV infection by HBsAg status leaving the other four HBV serological markers out of consideration, only patients with results of all five HBV serological markers were included in our study and the association between prior exposure to HBV (resolved HBV infection) and the development of extrahepatic cancer was investigated as well.^15^, ^16^, ^18^, ^45^, ^51^, ^62^ Therefore, more comprehensive and accurate information of the status of HBV infection could be provided and more credible results could be obtained in our study compared with others. As a case–control study, we chose individuals without malignant tumors who received routine medical checkups in the same hospital as non‐cancer controls to eliminate selection bias. Meanwhile, non‐cancer controls could more represent the general population than controls selected from hospitalized patients. Traditional propensity score matching was also conducted to balance covariates (including sex, age, BMI, smoking status, alcohol drinking status, diabetes mellitus, and family history of cancers) and reduce bias.

However, limitations still remained in our study. First, occult HBV infection which was defined as the absence of detectable HBsAg in serum and presence of HBV DNA in the liver (anti‐HBc, anti‐HBs, and anti‐HBe, are detected frequently in serum) might exist accompanied by HCV infection. As a possible confounding factor that may affect the role of HBV infection, we did not rule out the possibility of occult HBV infection. However, the prevalence of HCV was low in China (0.43%), and low prevalence of occult HBV infection has been reported as well.^63^, ^64^ As a limitation, participants with HIV or HCV infection were not excluded in our study due to information absence from cancer‐free controls. However, the prevalence of HIV and HCV was very low for included patients with cancers in our study (0.3% and 0.5%, respectively) and it was hard to determine the causal relationship between infection of viruses (HIV and HCV) and risk of gastric cancer, results of our study were still credible.^65^, ^66^, ^67^ Second, the number of non‐cancer controls was much larger than that of the cancer group before matching. Association was not observed between HBV infection and other cancers such as bile duct, small intestinal, and anal cancers which might be attributed to the small sample size and it was difficult to conclude cancers with low incidence. As esophageal squamous cell carcinoma is the predominant histological type, esophageal cancer was not included in our study. Thus, more cases were needed to be concerned with different types of cancers with low morbidity^68^ Third, as a well‐known infectious agent, H.pylori coinfection was not taken into consideration when analyzing the relationship between HBV infection and gastric cancer. However, evidence supporting the interaction between HBV and H. pylori was insufficient and a significant difference in H. pylori prevalence between the gastric cancer and any controls was not found in many previous studies.^11^, ^40^, ^42^, ^67^, ^69^, ^70^, ^71^ In addition, potential effects of confounding factors (included subjects’ nutritional status, dietary intakes, environmental exposure, socioeconomic status, access to health, educational level, etc.) could not be eliminated in our study by the absence of information which might cause decreased statistical power. Finally, as a more direct and accurate measure of active HBV infection compared with HBV serological markers, HBV DNA was not tested in our study. However, as an effective means to evaluate the status of HBV infection, HBV serological markers are still irreplaceable currently.

In summary, our study revealed that HBV infection was potentially associated with an increased risk of gastric cancer. However, because of the limited ability to establish a causal relationship for case–control study, large‐scale prospective cohort studies are urgent, and the latent mechanism about HBV extrahepatic carcinogenesis needs to be investigated as well.

## CONFLICTS OF INTEREST

None declared.

## Supporting information

Table S1‐S3

## Data Availability

Data are available upon reasonable request.
